# Supplementary material for: Polyketide synthase gene clusters in the ericoid mycorrhizal fungus Oidiodendron maius: genome-wide prediction, expression profiling, and functional characterization of knockout mutants
Source: Front Plant Sci. 2026 Sep 16;17:1924430. doi: 10.3389/fpls.2026.1924430 (PMC13623903; doi:10.3389/fpls.2026.1924430)
Supplement: Supplementary file 1 [file SupplementaryFile1.zip › Supplementary Materials/Supplementary Folder 1.zip!/Oidma1_AssemblyScaffolds_Repeatmasked/knownclusterblast/cluster50/jgi.p_Oidma1_207835_mibig_hits.html]

| MIBiG Protein | Description | MIBiG Cluster | MiBiG Product | % ID | % Coverage | BLAST Score | E-value |
| --- | --- | --- | --- | --- | --- | --- | --- |
| AAP03116.1 | putative\_carboxyesterase | BGC0000874 | Blasticidin\_biosynthetic\_gene\_cluster | 31 | 112.839506173 | 169 | 9.6e-42 |
| ABW96543.1 | putative\_type\_B\_carboxylesterase | BGC0000159 | Tautomycin\_biosynthetic\_gene\_cluster | 33 | 63.7037037037 | 128 | 1.4e-29 |
| ABI94378.1 | TMC\_biosynthetic\_enzyme\_L1 | BGC0000157 | Tautomycetin\_biosynthetic\_gene\_cluster | 32 | 71.3580246914 | 126 | 9.3e-29 |
| ABV91296.1 | putative\_type\_B\_carboxylesterase | BGC0000158 | Tautomycetin\_biosynthetic\_gene\_cluster | 32 | 71.3580246914 | 126 | 9.3e-29 |
| ACQ63629.1 | acyltransferase | BGC0000875 | Caprazamycin\_biosynthetic\_gene\_cluster | 34 | 56.049382716 | 119 | 1.1e-26 |
